# Comparative chloroplast genome analysis of five widespread species (*Zanthoxylum* L.) and development of molecular markers for their discrimination

**DOI:** 10.3389/fgene.2024.1495891

**Published:** 2024-12-24

**Authors:** Chong Sun, Huamin Liu, Yuan Guo, Xueqian Fu, Xinxin Zhu, Han Liu, Ning Tang, Zexiong Chen, Yiqing Liu, Xia Liu

**Affiliations:** ^1^ College of Horticulture and Gardening/Spicy Crops Research Institute, Yangtze University, Jingzhou, Hubei, China; ^2^ College of Smart Agriculture, Chongqing University of Arts and Sciences, Chongqing, China; ^3^ College of Life Science, Xinyang Normal University, Xinyang, Henan, China; ^4^ College of Biology and Food Engineering, Chongqing Three Gorges University, Chongqing, China

**Keywords:** zanthoxylum, complete chloroplast genome, phylogeny, species discrimination zanthoxylum, species discrimination, molecular markers

## Abstract

**Background:**

Zanthoxylum L., an important genus in the Rutaceae family, has great edible and medical values. However, the high degree of morphological similarity among *Zanthoxylum* species and the lack of sufficient chloroplast (cp) genomic resources have greatly impeded germplasm identification and phylogenetic analyses of *Zanthoxylum.*

**Methods:**

Here we assembled cp genomes of five widespread species (*Zanthoxylum bungeanum*, *Z. armatum*, *Z. nitidum*, *Z. ailanthoides* and *Z. piasezkii*) in China as a case study, comparative analysis of these assembled cp genomes.

**Results:**

Each of them, ranging from 157,231 to 158,728 bp, has a quadripartite structure. Except for one extra gene in *Z. piasezkii*, 132 genes were identified in each species, including 87 encode protein genes, 37 transfer ribose nucleic acid (tRNA) genes, and eight ribosomal RNA (rRNA) genes. Substantial variation was observed among these five cp genome sequences in the IR/SC boundary regions. Variation in insertions and deletions were observed in the cp genomes of the five species over three different intervals, and a large number of single-nucleotide polymorphism variants were detected in the *rps3*-*rpl22*-*rps19* region. Phylogenetic analysis of complete cp genome sequences revealed the evolutionary relationships among 23 *Zanthoxylum* species (29 samples).

**Conclusion:**

Comparative analysis revealed that *rps3*-*rpl22*-*rps19* is a highly variable divergent region in *Zanthoxylum* that could be developed as candidate markers for phylogenetic studies and species identification. This study identified a pair of molecular markers from hypervariable regions that can be used to distinguish between the five *Zanthoxylum* species and validated their utility. Overall, the results of this study provide new insights into the genetic breeding, germplasm exploration, and phylogeny of *Zanthoxylum* species.

## 1 Introduction

Ancestral populations of Chinese peppers inhabit the mountain regions in Shanxi, Henan, Hunan, Gansu, Hubei and Sichuan Provinces and are highly adapted to a cold and dry environment, which may indicate the presence of an important gene pool. However, wild populations are largely absent from marginal regions or have been replaced by cultivated populations (Zhang M. et al., 2017; Zeng, 2000). Therefore, the identification and evaluation of Chinese pepper genetic resources are important for their effective utilization and preservation.

Plant chloroplasts are the principal sites of photosynthesis and carbon fixation (Douglas, 1998). The genetic information in the chloroplast genomes contains valuable information for molecular evolution, phylogeny, and species identification (Zeng et al., 2017; Yang and Ji, 2017; Xu et al., 2018). Chloroplast genomes in angiosperms typically contain a quadripartite circular structure with one large copy (LSC), one small copy (SSC), and two copies of inverted repeats (IR) (Yang et al., 2014). In addition, chloroplast genomes can also be mapped rapidly, efficiently, and affordably using next-generation sequencing techniques.

There are several species of *Zanthoxylum* in the Rutaceae family, a group of shrub or arboreal plants found across Asia, Africa, Oceania, and North America (Huang, 1997). Among them, 45 species and 13 varieties are found in China, which is considered one of the major diversity centers for *Zanthoxylum* (Feng et al., 2020). Among these, five wild Chinese pepper resources are the most common in China, including *Zanthoxylum bungeanum* Maxim., *Zanthoxylum armatum* DC., *Zanthoxylum nitidum* (Roxb.) DC., *Zanthoxylum ailanthoides* Sied. et. Zucc. and *Zanthoxylum piasezkii* Maxim. The fruits of *Zanthoxylum* have been used as spices throughout eastern Asia for centuries. In traditional Chinese medicine, the roots, stems and leaves of many *Zanthoxylum* species are used to treat toothache, stomachache, neuralgia, rheumatic arthralgia, sore throat and snakebites (Yang et al., 2008). *Z. nitidum*, particularly, has a variety of physiological uses, and it even has anticancer and anti-inflammatory properties (Li and Wang, 2013).

Currently, majority of research on *Zanthoxylum* species is focused on its chemical components and pharmacological properties (Yang et al., 2008), and phylogenetic analysis using chloroplast markers including *trnL-trnF* and *matK* (Wang et al., 2016; Feng et al., 2017; Li et al., 2019), but chloroplast whole-genome comparison and phylogenetic analyses of *Zanthoxylum* species are less common. Phenotypes may be easily affected by environmental changes. For example, *Z. bungeanum* and *Z. nitidum* have different morphological types in different distribution areas (Huang, 1997; Qin et al., 2019). Phenotypic identification requires some special tissues and organs to identify, and sometimes it is difficult to accurately identify and classify. Therefore, molecular identification of *Zanthoxylum* species in China is needed. In this study, the complete chloroplast genomes of the five widespread *Zanthoxylum* species (*Z. bungeanum*, *Z. armatum*, *Z. nitidum*, *Z. ailanthoides* and *Z. piasezkii*) were sequenced, assembled and comparatively analyzed with those from fifteen previously published *Zanthoxylum* species. The genomes of the different species differ clearly at the molecular level and can be used to identify and divide them. Identifying repeated sequences and observing the overall structure of chloroplast genomes may help develop SSR markers that can be used to analyze the genetic diversity of *Zanthoxylum* species. In addition, by analyzing the entire chloroplast genome, we can better resolve deep-level relationships within the plant taxa in the future, which is of great significance for formal species authentication. Our findings will supply rich fundamental data on chloroplast genetic diversity and evolutionary relationships and contribute to *Zanthoxylum* species genetic resources for further research in this genus.

## 2 Materials and methods

### 2.1 Sampling, DNA extraction and sequencing

Fresh leaves of *Z. piasezkii*, *Z. bungeanum* and *Z. armatum* were collected from Sichuan Academy of Botanical Engineering, Neijiang City, Sichuan Province, China, and leaves of *Z. ailanthoides* and *Z. nitidum* were obtained from South China Botanical Garden in Guangzhou, China. Information of the samples is listed in Supplementary Table S1. Voucher specimens of the five *Zanthoxylum* species (voucher numbers: 18LCS, 18LHJ1, 19L-ZY1, 19L-LMZ, and 19L-CY1) were preserved in the herbarium of Chongqing University of Arts and Sciences, Chongqing, China. The fresh leaves were collected and dried with silica gel. Using an improved CTAB protocol (Allen et al., 2006), 100 mg leaf tissue was used to isolate the genome. After DNA isolation and quality testing, DNA fragments were used to build 350 bp short-insert libraries, which were then sequenced on the BGISEQ-500 using PE 150 bp (BGI, Shenzhen, China) following the manufacturer’s protocol.

### 2.2 Chloroplast genomes assembly and annotation

High-quality clean paired-end reads were mapped to the reference chloroplast of *Z. bungeanum* (accession number: NC_031386) obtained from GenBank using Bowtie 2 v.2.3.4.3 (Langmead and Salzberg, 2012) with default parameters. NOVOPlasty v.2.6.2 (Dierckxsens et al., 2017) used three coding gene sequences with the greatest coverage for *de novo* assembly of the chloroplast genome. Genes annotation and analysis were performed using the GeSeq (Tillich et al., 2017) and CPGAVAS2 (Shi et al., 2019) software. The tRNA genes were detected using tRNAscan-SE v2.0 (Chan and Lowe, 2019), and then manually adjusted and confirmed using Geneious v9.1.8 (Kearse et al., 2012). The circular genome map was drawn by Organellar GenomeDRAW tool (OG-DRAW) v.1.3.1 (Greiner et al., 2019) for further comparison of the gene order and content with default settings. A similar method was applied to the annotation of the other genomes obtained from GenBank for comparative analysis. The assembled complete plastome sequences of the five species have been submitted to GenBank with the accession numbers MW206785, MW206786, MW602887, MW602879, and MW478808.

### 2.3 Chloroplast genomes comparison and sequence variation analysis

Codonw software (Sharp and Li, 1987) was used to calculate the Relative Synonymous Codon Usage (RSCU) for each codon in each genome. These plastome junction regions were compared using the IRscope + online program (Amiryousefi et al., 2018) to determine the position of their junctions between IR, SSC, and LSC. Shuffle-LAGAN mode (Brudno et al., 2003) in mVISTA 2.0 (Frazer et al., 2004) was used to visualize divergent regions with the *Z. ailanthoides* (MW478808) genome as a reference. The nucleotide sequence polymorphism was determined using DnaSP v6.12.03 (Rozas et al., 2017) with a 15 bp step size and a 200 bp window length. The program REPuter (Kurtz et al., 2001) with adjusted parameters was used to identify long repeat sequences (forward, palindrome, complement and reverse repeats) in the five plastomes. The minimum repeat size was determined to be 25 bp, and the two repeat copies had greater than 90% similarity. SSRs with minimal repeat numbers of 8, 5, 3, 3, 3, and three were detected for mononucleotide, dinucleotide, trinucleotide, tetranucleotide, pentanucleotide, and hexanucleotide repeats, respectively, and were used in MISA v1.01 (Beier et al., 2017).

### 2.4 Phylogenetic analysis

The genome sequences of fifteen plastomes of the *Zanthoxylum* species were downloaded from the NCBI database, and the five newly assembled *Zanthoxylum* chloroplast genomes were used to analyze the phylogenetic relationships; *Phellodendron chinense* and *Tetradium ruticarpum* were set as the outgroups. The samples information is listed in Supplementary Table S2. Alignment was performed using MAFFT v7.0 with default parameters (Katoh and Standley, 2016), and then manual adjustment of multiple sequence alignment was performed with BioEdit software (Hall, 1999). PhyML v2.4.4 (Guindon and Gascuel, 2003) and MrBayes v3.2.6 (Ronquist and Huelsenbeck, 2003) were used to construct the phylogenetic relationships. Nucleotide substitution models were determined using jModelTest v2.1.1 (Posada, 2008). ML analyses were conducted using a GTR + I + G model with 1000 replicates.

### 2.5 Molecular markers for species discrimination: development and validation

Molecular markers were developed based on variable regions of cp genomes to discriminate among the five *Zanthoxylum* species using Geneious Prime software (Kearse et al., 2012). PCR amplification was conducted using a pair of primers designed using Geneious Prime (Kearse et al., 2012). PCR reactions and the thermal cycling conditions were followed as described in Chong et al. (2022). Finally, the PCR products were visualized using 1% agarose gel electrophoresis and subsequently subject to Sanger sequencing.

## 3 Results

### 3.1 The chloroplast genome structures of *zanthoxylum* species

**FIGURE 1:**
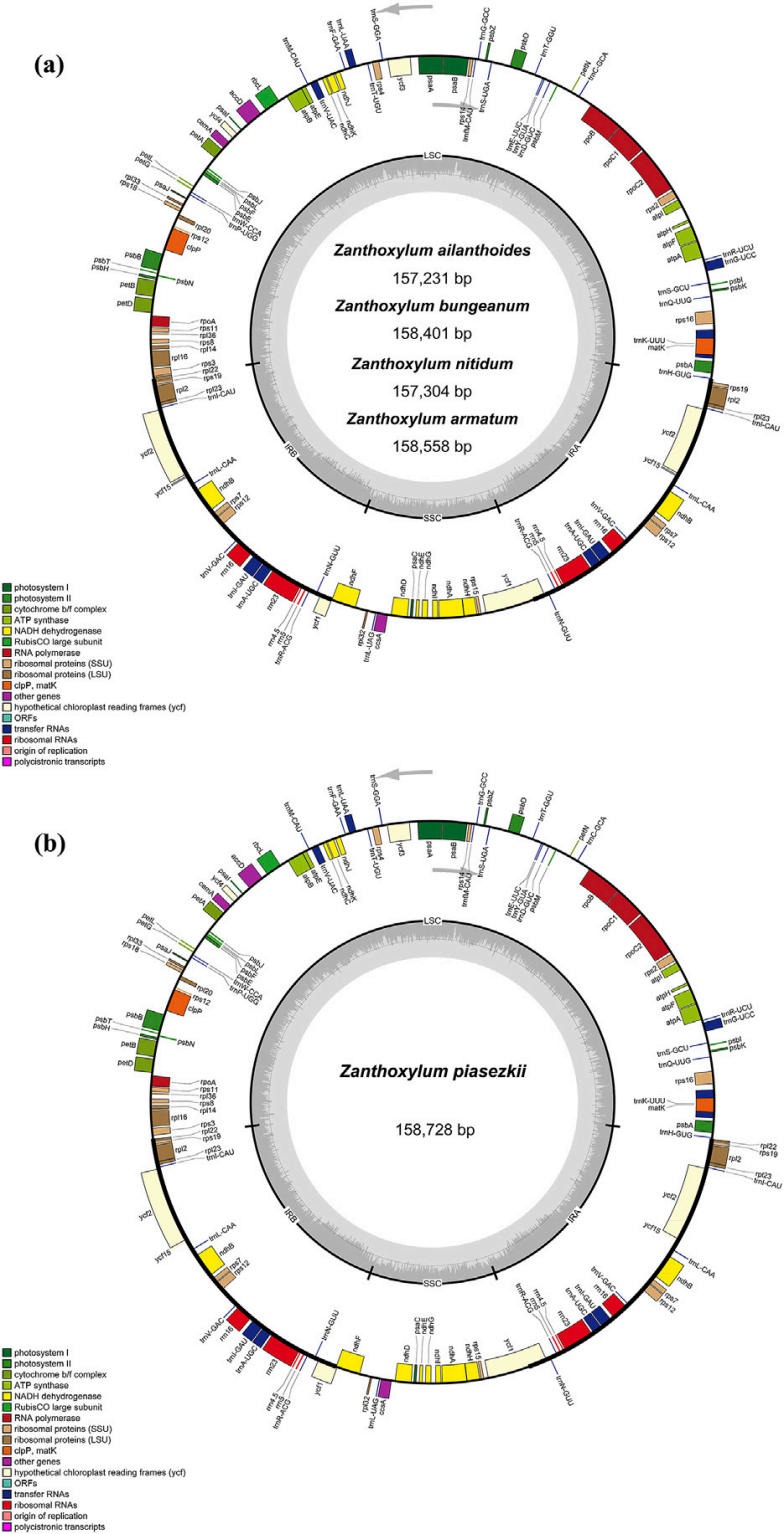
Gene map of the complete chloroplast genome of five *Zanthoxylum* species. Gene map of chloroplast genome of **(A)** *Zanthoxylum ailanthoides*, *Zanthoxylum bungeanum*, *Zanthoxylum nitidum*, *Zanthoxylum armatum*; **(B)** *Zanthoxylum piasezkii*. Genes in the circle are transcribed clockwise, while those outside are transcribed counter clockwise. The light gray inner circle and the dark gray shading in the inner circle corresponds to the AT and GC content, respectively. Genes belonging to different functional groups are shown with various colors.

Five *Zanthoxylum* species were sequenced, revealing a typical quadripatite structure, with a range in length from 157,231 (*Z. ailanthoides*) to 158,728 bp (*Z. piasezkii*) (Figure 1; Table 1). There are two inverted repeat (IRs, 26,408-27,651 bp) regions in the plastid genome, IRa and IRb, each separated by a large single-copy sequence (LSC, 84, 368–86, 122 bp) and a small single-copy sequence (SSC, 17, 603–18, 293 bp). There was a slight variation in the GC content of plastomes among the five species, ranging from 38.4% to 38.5% (Table 1). Most *Zanthoxylum* species possess 132 genes encoded by their plastomes, which include 87 protein-coding, 37 transfer RNA (tRNA) and eight ribosomal RNA (rRNA) genes (Figure 1A; Table 1; Table 2), while there were 133 genes (88 protein-coding genes) in *Z. piasezkii* (Figure 1B; Table 1; Table 2). The main difference was that there were two *rpl22* genes in *Z. piasezkii*, which were located on the IR side of the junction between IR and LSC. The length of *rpl22* in *Z. piasezkii* was relatively short, only 168 bp, while that in other four species were relatively long, ranging from 255 to 450 bp, which is only located across the LSC/IRb junction.

**TABLE 1:** The new sequenced and annotated complete chloroplast genomes of *Zanthoxylum* species.

Species	Accession No.	Plastome (bp)	LSC (bp)	SSC (bp)	IRs (bp)	Total genes	Protein-coding genes	tRNA genes	rRNA genes	Overall GC content (%)
*Z. bungeanum*	MW206786	158,401	85, 898	17, 611	27, 466	132	87	37	8	38.5
*Z. piasezkii*	MW206785	158,728	85, 918	17, 612	27, 599	133	88	37	8	38.4
*Z. armatum*	MW602887	158,558	85, 759	17, 603	27, 598	132	87	37	8	38.5
*Z. nitidum*	MW602879	157,304	84, 368	17, 634	27, 651	132	87	37	8	38.5
*Z. ailanthoides*	MW478808	157,231	86, 122	18, 293	26, 408	132	87	37	8	38.4

**TABLE 2:** Annotated gene classification of the chloroplast genome of *Zanthoxylum* species.

Category	Genes group	Genes name	Number
Self-replication	tRNA genes	trnH-GUG、trnK-UUU*、trnQ-UUG、trnS-GCU、trnG-UCC*、trnR-UCU、trnC-GCA、trnD-GUC、trnY-GUA、trnE-UUC、trnT-GGU、trnS-UGA、trnG-GCC、trnfM-CAU、trnS-GGA、trnT-UGU、trnL-UAA*、trnF-GAA、trnV-UAC*、trnM-CAU、trnW-CCA、trnP-UGG、trnI-GAU(2)*、trnL-CAA(2)、trnV-GAC(2)、trnI-GAU(2)、trnA-UGC(2)*、trnR-ACGc、trnN-GUU(2)、trnL-UAG	37
	rRNA genes	*rrn5(2)、rrn4.5(2)、rrn16(2)、rrn23(2)*	8
	DNA-dependent RNA polymerase	rpoC1*、rpoC2、rpoA、rpoB	4
	Ribosomal small subunit	rps16*、rps2、rps14、rps4、rps18、rps12(2)*、rps11、rps8、rps3、rps19(2)、rps15、rps7(2)	15
	Ribosomal large subunit	rpl33、rpl20、rpl36、rpl14、rpl16、rpl22(2)、rpl2(2)*、rpl23(2)、rpl32、	11/12
Photosynthesis	Photosystem I	psaA、psaB、psaC、psaI、pafI**、pafII、psaJ	7
	Photosystem II	*psbA、psbB、psbC、psbD、psbK、psbI、psbM、psbZ、psbJ、psbL、psbF、psbE、psbT、psbN、psbH*	15
	Cytochrome b/f complex	petN、petA、petL、petG、petB*、petD*	6
	ATP synthase	atpE、atpB、atpA、atpF*、atpH、atpI	6
	Protease	clpP**	1
	Large subunit of rubisco	*rbcL*	1
	NADH dehydrogenase	ndhJ、ndhK、ndhC、ndhB(2)*、ndhF、ndhD、ndhE、ndhG、ndhI、ndhA*、ndhH	12
Others	Maturase	*matK*	1
	Envelope membrane protein	*cemA*	1
	Subunit of acetyl-CoA carboxylase	*accD*	1
	Cytochrome c synthesis	*ccsA*	1
Function unknown	Open reading frames	*ycf1(2)、ycf2(2)、ycf15(2)*	6

Note: *Gene contains one intron; **Gene contains two introns; (2) indicates with two copies of the gene.

Among these genes, nine protein-coding genes (*rps19*, *rps7*, *rpl2*, *rpl23*, *rpl22*, *ycf2*, *ycf15*, *ndhB*, and *ycf1*), seven tRNA genes (*trnR-ACG*, *trnN-GUU*, *trnA-UGC*, *trnV-GAC*, *trnL-GAU*, *trnL-CAA*, and *trnL-CAU*) and four rRNA genes (*rrn5*, *rrn16*, *rrn4.5*, and *rrn23*) were duplicated in the IR regions. Additionally, 19 genes, including 13 protein-coding genes and six tRNA genes, had two exons, while four protein-coding genes (*pafI*, *clpP1*, and two *rps12*) had three exons (Supplementary Table S3).

**FIGURE 2:**
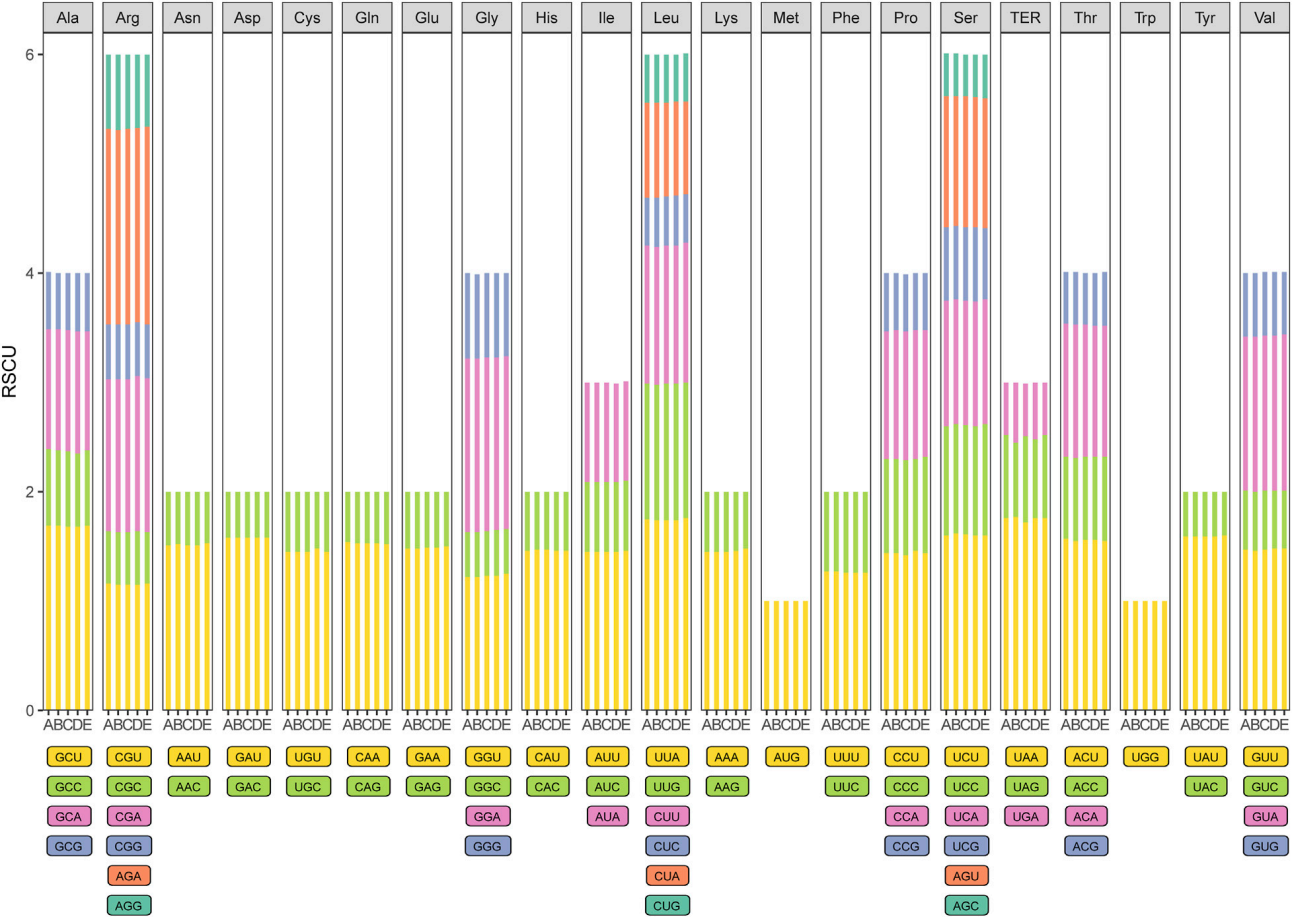
Codon content of 20 amino acid and stop codons in all protein-coding genes of the five chloroplast genomes. The histogram from the left-hand side of each amino acid shows codon usage within *Zanthoxylum* (From left to right: A: *Zanthoxylum bungeanum*; B: *Zanthoxylum piasezkii*; C: *Zanthoxylum armatum*; D: *Zanthoxylum nitidum*; E: *Zanthoxylum ailanthoides*).

All of the protein-coding genes were composed of 25,825–26,512 codons in the chloroplast genomes of the five species of *Zanthoxylum* (Figure 2; Supplementary Table S4). Among these codons, leucine, arginine and serine represent the most abundant amino acids, whereas methionine (1.10%–1.15%) has the lowest abundance. Based on the relative synonymous codon usage (RSCU) statistical analysis all amino acids have more than one synonymous codon, except for methionine (AUG) and tryptophan (UGG) (RSCU = 1). Moreover, half of the codons had RSCU > 1, and most of those (29/31, 93.5%) ended with A or U. The rest of the codons had RSCU < 1, and most of those (28/31, 90.3%) ended with G or C (Supplementary Table S4).

### 3.2 Comparative genomic divergence and hotspots regions

**FIGURE 3:**
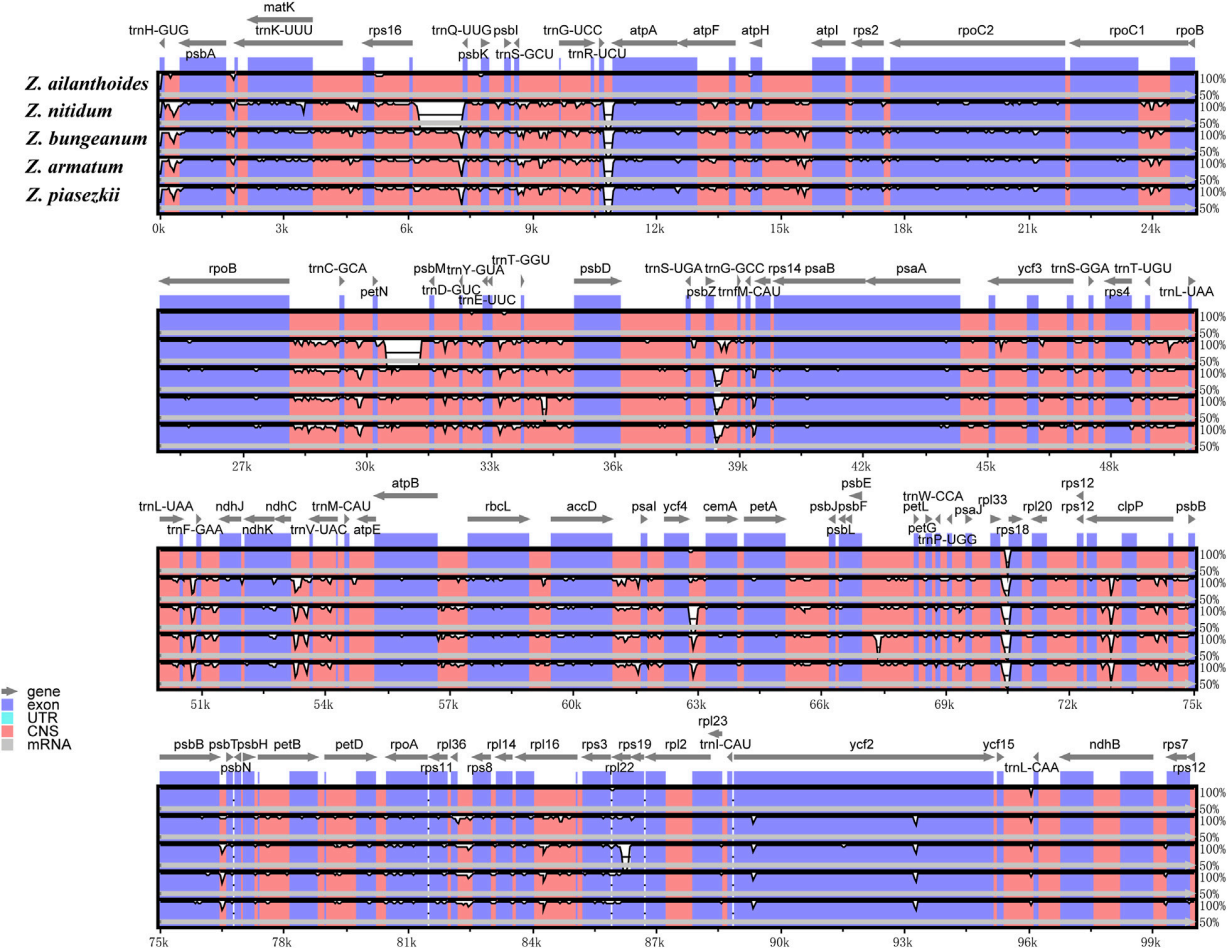
Comparison of the five chloroplast genomes of *Zanthoxylum* by using mVISTA. Grey arrows indicate genes orientation, and thick black lines above the alignment indicate IR position. Plots were based on a 70% identity cut-off, and the Y-scale represents the percent identity from 50% to 100%. The color-coded of genome regions refer as protein-coding (exon; blue), conserved non-coding sequences (CNS; pink), and white peaks represent divergences in the chloroplast genomes.

**FIGURE 4:**
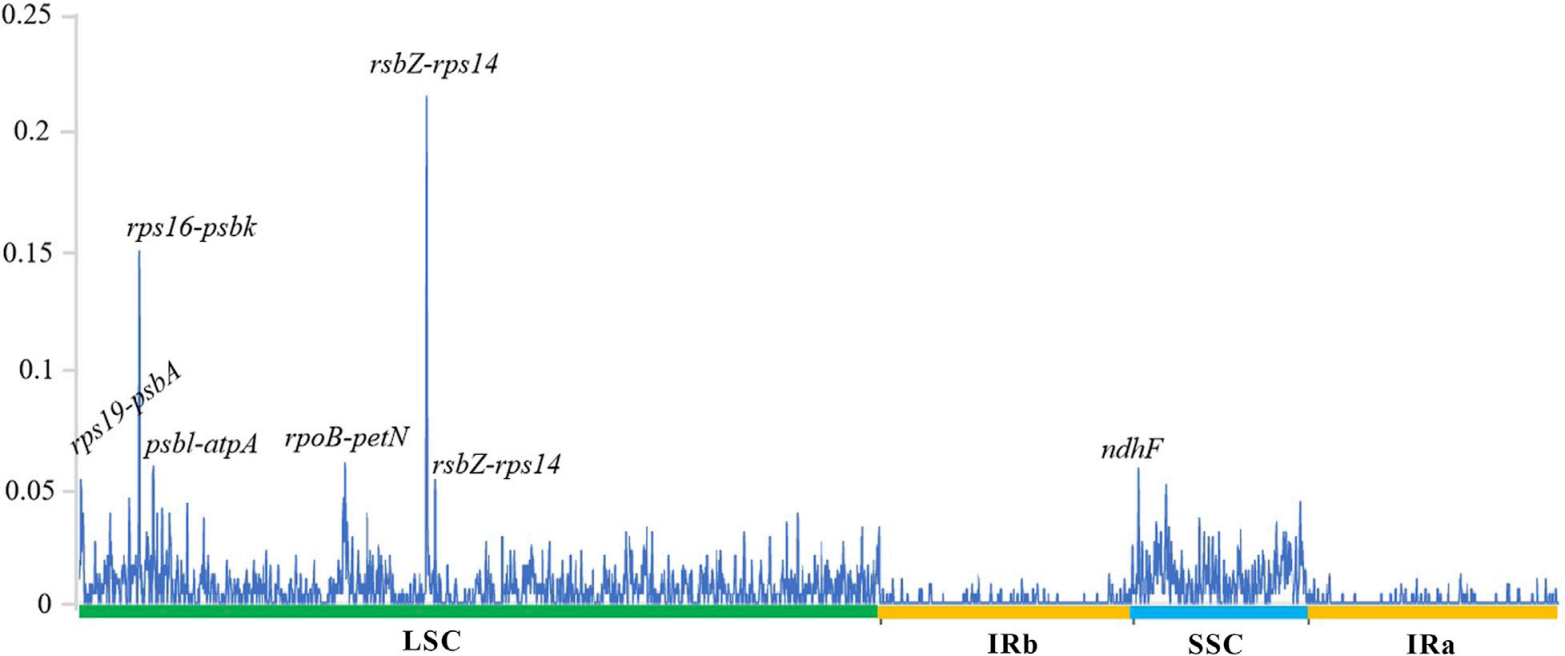
Comparison of the nucleotide variability (Pi) values among the five species chloroplast genomes. The *X*-axis indicates the genes with high Pi values, the *Y*-axis indicates the Pi values.

The sequence divergence of the cpDNA of five species (*Z. nitidum, Z. bungeanum, Z. armatum*, *Z. ailanthoides* and *Z. piasezkii*) was drawn by mVISTA, with *Z. armatum* (MW602887) as a reference (Figure 3). *Z. ailanthoides’* entire chloroplast sequence revealed sequence variation different from the four other species. The comparison among the five cpDNAs showed that the intensity of variation in the IR region was low, and the most variation was observed in the LSC regions and in the combined sites of the IR and LSC regions. Furthermore, the sequence divergence among the five entire cp genomes was evaluated for the nucleotide diversity (Pi) value (Figure 4). In terms of rps19-psbA, *rps16-psbK*, p*sbL-atpA*, *rpoB-petN*, *rsbZ-rps14*, and *ndhF* displayed the highest Pi values, whereas *rsbZ-rps14* displayed the most variability. These divergent areas might serve as molecular markers for identifying *Zanthoxylum* plants and systematic evolution analyses.

### 3.3 IR contraction and expansion

**FIGURE 5:**
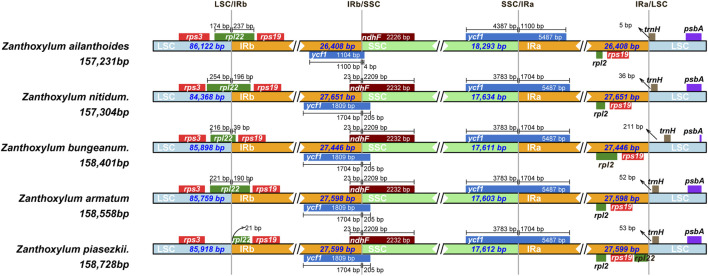
Comparison of the junctions of the LSC, SSC and IR regions in the chloroplast genomes of five *Zanthoxylum* species.

The IR regions of the five chloroplast genomes ranged in size from 26,408 bp (*Z. ailanthoides*) to 27,651 bp (*Z. nitidum*) (Table 1; Figure 1). We compared the IR borders in five widespread *Zanthoxylum* species, and the results showed that the IR junction regions showed slight changes (Figure 5). The *rps3* and *rps19* genes were fully located in the LSC and IR regions for five *Zanthoxylum* species, respectively. It is worth noting that the *rpl22* gene was situated in the LSC/IRb border for *Z. ailanthoides, Z. nitidum*, *Z*. *bungeanum*, and *Z. armatum*, while the *rpl22* gene of *Z. piasezkii* was truncated in the IRa and IRb regions (Figure 5). The *ycf1* was located in the SSC/IR borders, and 5,487 bp of this gene extended into the IRa region. The *ycf1* gene was situated in IRb/SSC boundary having equal size 205 bp expanded into the SSC region for *Z. nitidum*, *Z. bungeanum*, *Z. armatum*, and *Z. piasezkii,* with only four bp expanded into the SSC region for *Z. ailanthoides.* The photosynthetic *ndhF* gene was situated in the SSC region in *Z. ailanthoides*, and 23 bp of this gene extended from the SSC region to the IRb region in the *Z. nitidum*, *Z. piasezkii*, *Z. bungeanum*, and *Z. armatum* cp genomes. The *rpl2* gene was located in the IRa region in the cp genomes of the five samples, the *trnH* sequences gaps in the LSC region were 5 bp, 36 bp, 211 bp, 52 bp, and 53 bp away from the IRa/LSC junction.

### 3.4 Analyses of repeat structure and simple sequence repeats

**FIGURE 6:**
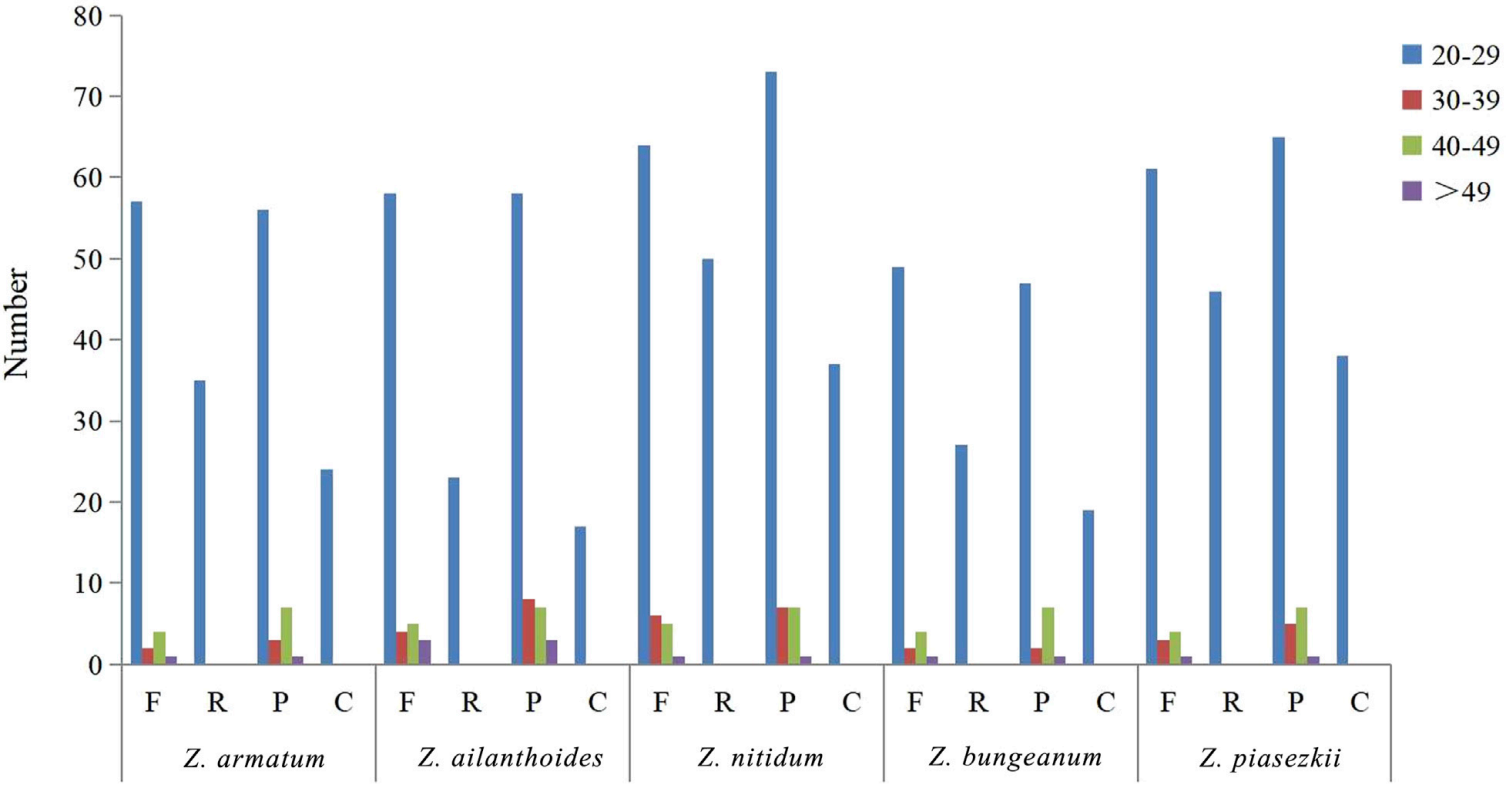
Repeat sequences in five chloroplast genomes. F, R, P, and C indicate forward, reverse, palindrome, and complement repeat types, respectively. Different colors are used to indicate repeats with different lengths.

**FIGURE 7:**
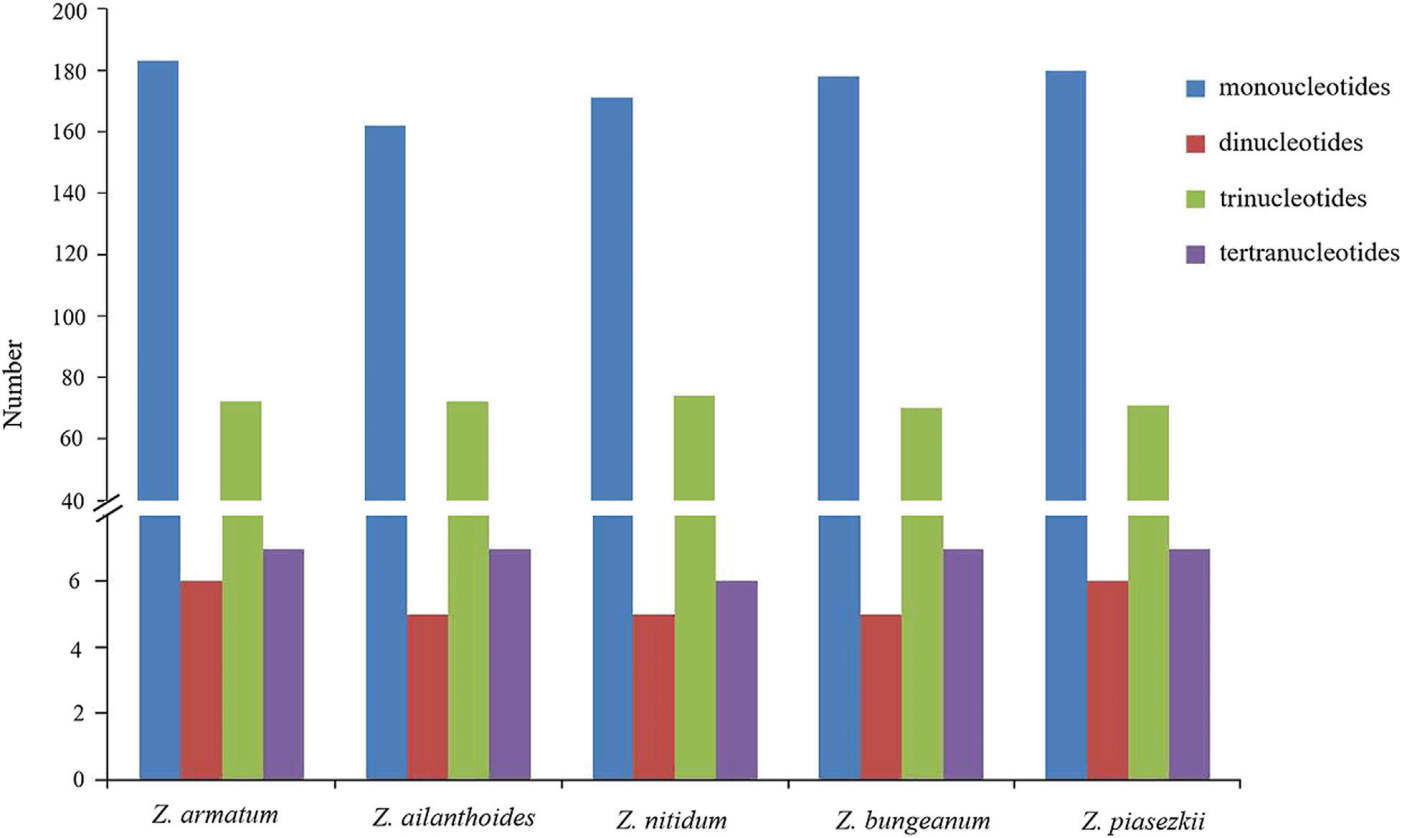
Analysis of simple sequence repeats (SSRs) in five *Zanthoxylum* chloroplast genomes.

For the repeat structure analysis in the five chloroplast genomes (Figure 6 and Supplementary Table S5), palindromic repeats were not found in all five species. Most repeats were between 20 and 39 bp in length, while the longest forward repeats were 73 bp in length and found in the LSC region in the plastidial genomes of the five species of *Zanthoxylum*. The total number of SSRs was also identified in the chloroplast genomes of the five species (Figure 7; Supplementary Table S6). Only four types of SSRs (mononucleotide, dinucleotides, trinucleotides, tetranucleotides repeat motifs) were identified in the five species. Most of these SSRs had mononucleotide repeats, with A/T repeats being the most common, with 97.1% and 96.7% found in *Z. nitidum* and *Z. armatum*, respectively, while *Z. nitidum*, *Zanthoxylum pinasezkii* and *Z. bungeanum* are all characterized by AT/TA repeats in their dinucleotide repeat motifs (all 100%). The five species of *Zanthoxylum* all comprised AAAT/ATTT and ACAT/ATGT repeats.

### 3.5 Phylogenetic relationship analyses

**FIGURE 8:**
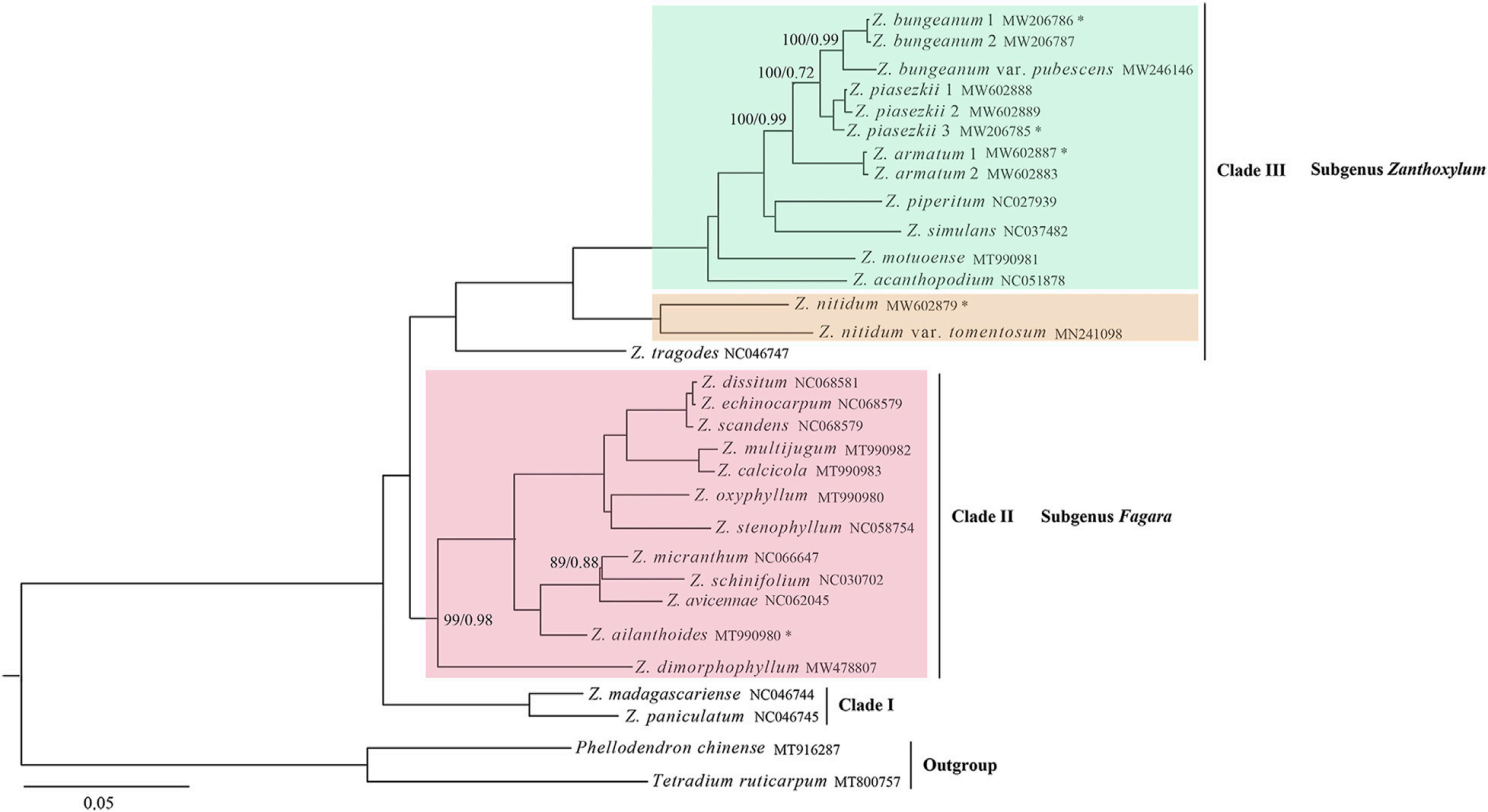
Phylogenetic relationships of the 29 members within *Zanthoxylum* based on the whole cp genomes using ML and BI analysis. Marked with an asterisk corresponds to the newly sequenced species in this study. *Phellodendron chinense* and *Tetradium ruticarpum* were used as the outgroups. Numbers above nodes are support values (ML bootstrap support values and Bayesian posterior probability values), no value is displayed on the node indicates the support rate is 100/1.00.

The maximum likelihood (ML) and Bayesian inference (BI) methods were used to construct a phylogenetic tree using 29 *Zanthoxylum* plastid genome sequences (160,430 bp), and *P. chinense* and *T. ruticarpum* were used as outgroups. The phylogenetic trees constructed by the two methods were similar topology with only slight differences in the support values in some branches (Figure 8). All *Zanthoxylum* species formed a high-resolution clade. *Zanthoxylum paniculatum* and *Zanthoxylum madagascariense* were in the basal position and clustered together to form a single branch and then sisters to twelve species of the subgenus *Fagara* of *Zanthoxylum*. *Zanthoxylum tragodes* is the sister species of eight *subgenus Zanthoxylum* species (14 samples). However, it is worth noting that *Z. nitidum* and *Z. nitidum* var. *tomentosum* belongs to *subgenus Fagara* based on the record in the Flora of China (Huang, 1997), but rather than being grouped together *subgenus Fagara* branch, they were sister to *subgenus Zanthoxylum* species.

### 3.6 Molecular marker for species discrimination

**FIGURE 9:**
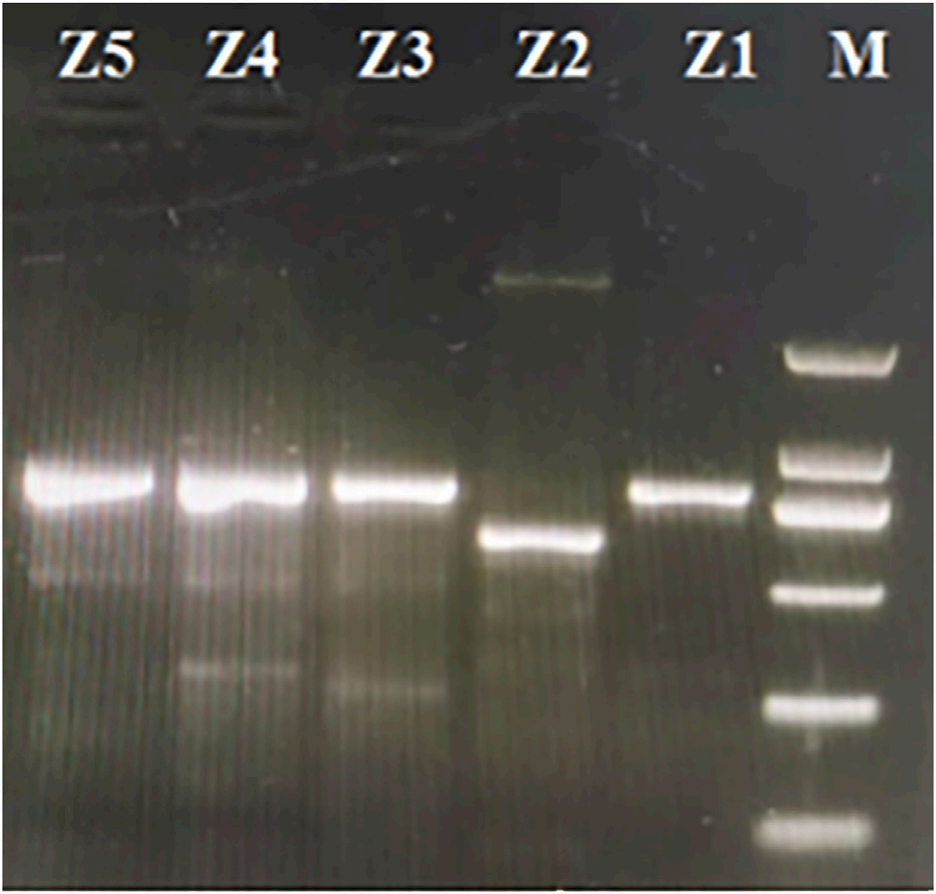
Gel electrophoresis of the amplification products using the designed primers. Lane M is the DL2000 marker. The lanes from right to left (marked Z1, Z2, Z3, Z2, and Z5) correspond to the products amplified from *Zanthoxylum nitidum*, *Zanthoxylum bungeanum*, *Zanthoxylum ailanthoides*, *Zanthoxylum piasezkii* and *Zanthoxylum armatum*, respectively.

**FIGURE 10:**
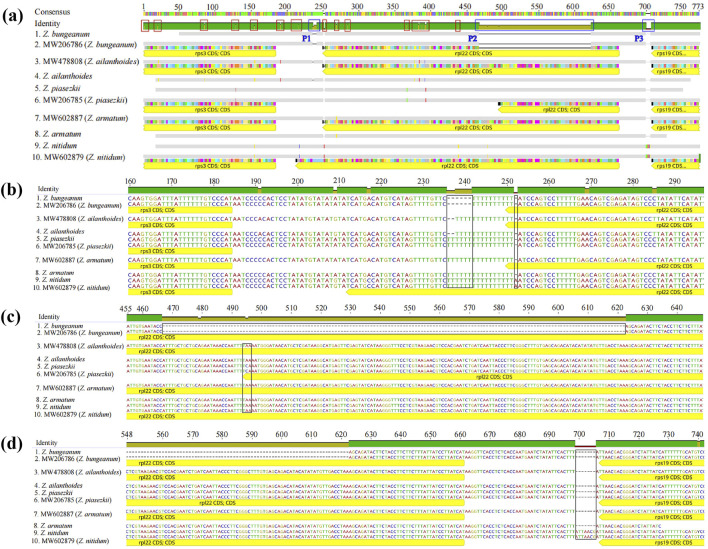
Sequence alignment of PCR products for the five *Zanthoxylum* species. **(A)** Overall view of the alignment of the PCR products and assembly sequences. 1, 4, 5, 8, and 9 correspond to the sequences from the PCR products, and 2, 3, 6, 7, and 10 correspond to the assembled sequences. **(B, C, D)** show larger versions of the blue frames from left to right (marked P1, P2, and P3, respectively) in **(A)**; The red frame indicates the 18 SNPs in the *rps3*-*rpl22*-*rps19* region, and the black frame indicates the area of variation.

The specific primer pairs were designed for the conserved sequences on either side of the variation regions according to the cp genomes analysis to develop molecular markers that could be used to discriminate among these five *Zanthoxylum* species. The target fragments were amplified in the five *Zanthoxylum* samples. These primer pairs could be used to distinguish *Z. bungeanum* by PCR (Figure 9), and the PCR products of the five *Zanthoxylum* species were sequenced. The genome sequence obtained by high-throughput sequencing was compared with the sequence obtained by PCR product sequencing (Figure 10); variation in indels was observed over three intervals in the *rps3*-*rpl22*-*rps19* region (Figure 10a). The P1 mutation region was located in the *rps3*-*rpl22* region, the P2 mutation region was located in the *rpl22* genes, and the P3 mutation region was located in the *rpl22*-*rps19* region; the P2 mutation region was the largest (Figure 10; Figure 10A). This region was 156 bp smaller in the *Z. bungeanum* cp genome than the other four samples cp genomes, indicating that it can be used as a marker for the identification of *Z. bungeanum* (Figure 9). A base mutation was present in *Z. piasezkii* in the *rpl22* gene in the P2 region, but this mutation was not present in other species (Figures 10A,C); this base mutation resulted in the change of a T base in the middle of the TTA codon to a G base, which resulted in the premature termination of the *rpl22* gene. In the P1 region (Figure 10B), *Z. bungeanum* had a 7 bp deletion that was absent in the other species, and *Z. ailanthoides* had a three bp deletion that was absent in the other samples, with the exception of *Z. bungeanum*, indicating that the region could be developed markers for identifying *Z. bungeanum* and *Z. ailanthoides*. A single base insertion (A) was present in the *rpl22* gene of *Z. nitidum* relative to the *rpl22* genes in other species (Figure 10B), and this insertion resulted in a frameshift mutation in the *rpl22* gene of *Z. nitidum* and thus an increase in its length. This site could be used for the identification of *Z. nitidum*. A 7 bp insertion present in *Z. nitidum* was also observed in the other three species in the P3 mutation region (Figure 10D), which could be used as a marker for the identification of *Z. nitidum*.

## 4 Discussion

### 4.1 The features of *zanthoxylum* species chloroplast genomes

This study examined the chloroplast genomes of five *Zanthoxylum* species. It was found that all five species had a typical structure and order of characteristics, which were consistent with the characteristics of angiosperms (He et al., 2017). There was less divergence among the coding regions than among the noncoding regions, and the inverted repeat regions (IRs) were more conserved than the single-copy (SC) regions. Similar results have also been found in the chloroplast genomes of other genera, including *Saposhnikovia* (Bao et al., 2022), *Gynostemma* (Zhang X. et al., 2017), *Fritillaria* (Yu et al., 2018), *Rehmannia* (Zeng et al., 2017), and *Aconitum* (Park et al., 2017). *Zanthoxylum* chloroplast genomes exhibited a lower GC content than AT content, a phenomenon seen in other angiosperm chloroplast genomes (Yang et al., 2010; Asaf et al., 2017; Raubeson et al., 2007). In addition, the results showed that the GC content was highest in IR regions, possibly due to the large quantities of rRNA present there.

SSRs (simple sequence repeats) are widely used to study plant populations, identify species, and infer their evolutionary history (Provan, 2000; Flannery et al., 2006). Li et al. (Li et al., 2019) developed SSRs derived from the chloroplast genome (cpSSRs) to analyze the genetic diversity among *Zanthoxylum* species*.* In our study, 246–268 SSRs were identified in the five plastid genomes (Figure 7; Supplementary Table S6). The number of poly(C)/(G) SSRs in the *Zanthoxylum* cp genome is much lower than that of poly (A)/(T) (Supplementary Table S6), which is consistent with previous findings (Zhao et al., 2021). In addition, *Zanthoxylum*’s phylogenetic relationships were revealed using effective markers designed based on the five chloroplast genomes*.* Overall, *Zanthoxylum* species will benefit from these newly identified cpSSR markers, contributing to understanding their diversity, genetic structure, and differentiation in the future.

### 4.2 IR expansion and contraction

Border contractions and expansions within the IR region, a dominant area for changes in the length of the chloroplast genomes and the shifts in the IR region borders, are common evolutionary events (Kode et al., 2005; Raubeson et al., 2007; Yao et al., 2015), are prevalent in many species, and play essential roles in plant evolution (Zhang et al., 2013; Wang et al., 2008). Many reports have indicated that IR regions are the most conserved regions of the chloroplast genome in most plant species (Daniell et al., 2016), but some studies have also reported that many chloroplast genome sequences in most plant species are rearranged, including the inversions and reinversions in the LSC and SSC regions (Doyle et al., 1992; Palmer et al., 1987; Cosner et al., 1997). In our study, the length of the chloroplast genomes of the five *Zanthoxylum* species (157,231-158,728 bp) did not change significantly which is consistent with the results reported by other researchers (Zhao et al., 2021). Although the IR junction regions of the five *Zanthoxylum* species were not significantly different, our results showed that apart from the photosynthetic *ndhF* genes of subgenus *Zanthoxylum*, which exhibited an identical (23 bp) distance from the SSC to the IRb regions for *Z. bungeanum, Z. armatum*, and *Z. piasezkii* (Figure 5), the *ndhF* genes of *Z. nitidum* also has this characteristic, which is different from previous research (Cosner et al., 1997), it may be related to not including the *Z. nitidum* sample in the previous study. The *trnH* sequence gaps in LSC have significantly different distances away from the IRa/LSC border in the five *Zanthoxylum* chloroplast genomes. Hence, the *psbA-trnH* intergenic spacer region was selected as a candidate DNA barcode sequence to distinguish similar species (Yao et al., 2009) and even at the family level (Degtjareva et al., 2012; Aldrich et al., 1988). Therefore, the *psbA-trnH* sequence region to develop DNA barcoding for *Z. nitidum, Z. bungeanum, Z. armatum*, and *Z. piasezkii*.

### 4.3 Phylogeny analysis

Chloroplast genome sequencing, especially the application of next-generation sequencing, has become simpler and easier with its continuous development (Xu et al., 2020). Currently, researchers have widely used the entire chloroplast genome sequence to conduct evolution, classification, and phylogenetic studies of angiosperms (Borsch and Quandt, 2009; Dong et al., 2012; Tong et al., 2016). Our study included 23 *Zanthoxylum* species with robust phylogenetic relationships based on ML and BI. The results showed that almost all relationships inferred from chloroplast genome data were generally highly supported based on the two methods (Figure 8). *Zanthoxylum* species were all clustered together to form a single clade with high resolution. It is noteworthy that *Z. nitidum*, *Z. nitidum* var. *tomentosum* in subgenus *Fagara* and *Z. tragodes* clustered together to form a single clade and sister to other subgenus *Zanthoxylum* species, supported *Z. nitidum* belonging to subgenus *Zanthoxylum* instead of subgenus *Fagara*, which showed some differences from the record in the Flora of China (Huang, 1997). However, the English version of the Flora of China (Zhang et al., 2008) did not mention the subgeneric classification of the genus *Zanthoxylum*. Actually, the morphological taxonomy of *Z. nitidum* remains controversial due to the abundance of phenotypic variations (Qin et al., 2019), morphological boundaries between varieties or types within *Z. nitidum* species should be re-evaluated (Qin et al., 2019). Hence, the accurate taxonomic delimitation of *Z. nitidum* remains to be further study with more substantial evidence based on more samples, genomic data, and phenotypic traits (Qin et al., 2022). In addition, *Z. paniculatum* and *Z. madagascariense* were in the basal position clustered together to form a single branch and then were sisters to other *Zanthoxylum* species. The two species may suggest a new subgenus. The traditional classification systems of *Zanthoxylum* that rely solely on external forms (petals and calyx) may be imperfect. Therefore, the existing classification of the genus *Zanthoxylum* can be further researched and updated by including more samples in the future. Overall, our research provides robust phylogenetic relationships of *Zanthoxylum* based on the entire chloroplast genome, which might provide insights into the genetic diversity, molecular breeding, and plastome evolution of the genus *Zanthoxylum.*

### 4.4 Molecular marker development based on *zanthoxylum* chloroplast genomes

A large number of indels (3 indel) and SNP sites (17 SNPs) were observed in the *rps3-rpl22-rps19* region (Figure 10A), this highly variable region of the cp genomes can be used as a marker for the identification of *Zanthoxylum* species. We designed specific primer pairs (F: GAG​CAA​TTC​CCT​CAA​CAC​CG; R: GGG​AGA​ATT​TGC​GCC​CAC​TA) for the different regions of these five *Zanthoxylum* species. The target fragments were amplified in all five *Zanthoxylum* samples. This DNA barcode developed in this study could be used to distinguish these five *Zanthoxylum* species (Figures 9, 10). Therefore, *rps3-rpl22-rps19* region are proposed as barcodes for rapid and accurate identification of *Zanthoxylum* species.

## 5 Conclusion

In this study, we analyzed five species that are widely distributed and cultivated in China. The results showed that the genome length and gene content of the genus *Zanthoxylum* were comparatively conserved, while the IR-SC boundary regions were variable between the chloroplast genomes of the five *Zanthoxylum* species. The whole *rpl22* gene in the *Z. piasezkii* species was different from other *Zanthoxylum* species and could be used as a marker to identify *Z. piasezkii*. We identified SSR sites and seven variable regions that may be used to develop tools to study *Zanthoxylum* species in the future. A phylogenetic tree was constructed with the whole chloroplast genome to better understand the genetic relationships of the *Zanthoxylum* species. This result supports *Z. nitidum* belongs to subgenus *Zanthoxylum* instead of subgenus Fagara, which seems to suggest that the earlier intrageneric classifications need to be further refined. As to whether *Zanthoxylum* should be further divided into two subgenera, we will include more samples for further study in our future research. In brief, our research provides extensive genetic resources for studying the chloroplast genome of *Zanthoxylum* and provides valuable reference information for species identification and conservation.

## Data Availability

The datasets presented in this study can be found in online repositories. The names of the repository/repositories and accession number(s) can be found in the article/Supplementary Material.
