# Supplementary material for: Comparative chloroplast genome analysis of five widespread species (Zanthoxylum L.) and development of molecular markers for their discrimination
Source: Front Genet. 2024 Dec 24;15:1495891. doi: 10.3389/fgene.2024.1495891 (PMC11703814; doi:10.3389/fgene.2024.1495891)
Supplement: Supplementary file 1 [file Table3.doc]

Table S3 Genes containing exons and introns in the chloroplast genome of *Zanthoxylum* species

| Taxon | Gene | Location | Exon I  (bp) | Intron I  (bp) | Exon II  (bp) | Intron II (bp) | Exon IIII (bp) |
| --- | --- | --- | --- | --- | --- | --- | --- |
| *Z. bungeanum* | *trnK-UUU* | LSC | 37 | 2506 | 35 | - | - |
|  | *rps16* | LSC | 40 | 898 | 227 | - | - |
|  | *trnG-UCC* | LSC | 23 | 749 | 48 | - | - |
|  | *atpF* | LSC | 145 | 797 | 416 | - | - |
|  | *rpoC1* | LSC | 432 | 799 | 1611 | - | - |
|  | *pafI* | LSC | 124 | 737 | 230 | 796 | 153 |
|  | *trnL-UAA* | LSC | 35 | 552 | 50 | - | - |
|  | *trnV-UAC* | LSC | 39 | 595 | 35 | - | - |
|  | *rps12** | LSC | - | 538 | 232 | - | 26 |
|  | *clpP1* | LSC | 71 | 872 | 292 | 594 | 228 |
|  | *petB* | LSC | 6 | 754 | 642 | - | - |
|  | *petD* | LSC | 8 | 739 | 475 | - | - |
|  | *rpl16* | LSC | 9 | 1038 | 399 | - | - |
|  | *rpl2* | IR | 391 | 693 | 434 | - | - |
|  | *ndhB* | IR | 777 | 681 | 756 | - | - |
|  | *trnI-GAU* | IR | 37 | 960 | 35 | - | - |
|  | *trnA-UGC* | IR | 38 | 803 | 35 | - | - |
|  | *ndhA* | SSC | 553 | 1155 | 539 | - | - |
| *Z.ailanthoides* | *trnK-UUU* | LSC | 37 | 2506 | 35 | - | - |
|  | *rps16* | LSC | 40 | 894 | 227 | - | - |
|  | *trnG-UCC* | LSC | 23 | 740 | 48 | - | - |
|  | *atpF* | LSC | 145 | 796 | 416 | - | - |
|  | *rpoC1* | LSC | 432 | 777 | 1611 | - | - |
|  | *pafI* | LSC | 124 | 735 | 230 | 794 | 153 |
|  | *trnL-UAA* | LSC | 35 | 546 | 50 | - | - |
|  | *trnV-UAC* | LSC | 39 | 593 | 35 | - | - |
|  | *rps12** | LSC | - | 539 | 232 | 26 | - |
|  | *clpP1* | LSC | 71 | 829 | 292 | 646 | 228 |
|  | *petB* | LSC | 6 | 754 | 642 | - | - |
|  | *petD* | LSC | 8 | 738 | 475 | - | - |
|  | *rpl16* | LSC | 9 | 1049 | 399 | - | - |
|  | *rpl2* | IR | 391 | 693 | 434 | - | - |
|  | *ndhB* | IR | 777 | 681 | 756 | - | - |
|  | *trnI-GAU* | IR | 37 | 962 | 35 | - | - |
|  | *trnA-UGC* | IR | 38 | 803 | 35 | - | - |
|  | *ndhA* | SSC | 553 | 1151 | 539 | - | - |
| *Z. nitidum* | *trnK-UUU* | LSC | 37 | 2532 | 35 | - | - |
|  | *rps16* | LSC | 40 | 901 | 227 | - | - |
|  | *trnG-UCC* | LSC | 23 | 752 | 48 | - | - |
|  | *atpF* | LSC | 145 | 787 | 416 | - | - |
|  | *rpoC1* | LSC | 432 | 785 | 1611 | - | - |
|  | *pafI* | LSC | 124 | 738 | 230 | 816 | 153 |
|  | *trnL-UAA* | LSC | 35 | 552 | 50 | - | - |
|  | *trnV-UAC* | LSC | 39 | 595 | 35 | - | - |
|  | *rps12** | LSC | - | 540 | 232 |  | 26 |
|  | *clpP1* | LSC | 71 | 873 | 292 | 610 | 228 |
|  | *petB* | LSC | 6 | 756 | 642 | - | - |
|  | *petD* | LSC | 8 | 741 | 475 | - | - |
|  | *rpl16* | LSC | 9 | 1042 | 399 | - | - |
|  | *rpl2* | IR | 391 | 693 | 434 | - | - |
|  | *ndhB* | IR | 777 | 681 | 756 | - | - |
|  | *trnI-GAU* | IR | 37 | 962 | 35 | - | - |
|  | *trnA-UGC* | IR | 38 | 803 | 35 | - | - |
|  | *ndhA* | SSC | 553 | 1149 | 539 | - | - |
| *Z. armatum* | *trnK-UUU* | LSC | 37 | 2506 | 35 | - | - |
|  | *rps16* | LSC | 40 | 898 | 227 | - | - |
|  | *trnG-UCC* | LSC | 23 | 747 | 48 | - | - |
|  | *atpF* | LSC | 145 | 797 | 416 | - | - |
|  | *rpoC1* | LSC | 432 | 803 | 1611 | - | - |
|  | *pafI* | LSC | 124 | 737 | 230 | 795 | 153 |
|  | *trnL-UAA* | LSC | 35 | 552 | 50 | - | - |
|  | *trnV-UAC* | LSC | 39 | 596 | 35 | - | - |
|  | *rps12** | LSC | - | 538 | 232 | - | 26 |
|  | *clpP1* | LSC | 71 | 872 | 292 | 594 | 228 |
|  | *petB* | LSC | 6 | 753 | 642 | - | - |
|  | *petD* | LSC | 8 | 739 | 475 | - | - |
|  | *rpl16* | LSC | 9 | 1039 | 399 | - | - |
|  | *rpl2* | IR | 391 | 693 | 434 | - | - |
|  | *ndhB* | IR | 777 | 681 | 756 | - | - |
|  | *trnI-GAU* | IR | 37 | 960 | 35 | - | - |
|  | *trnA-UGC* | IR | 38 | 803 | 35 | - | - |
|  | *ndhA* | SSC | 553 | 1151 | 539 | - | - |
| *Z. piasezkii* | *trnK-UUU* | LSC | 37 | 2505 | 35 | - | - |
|  | *rps16* | LSC | 40 | 897 | 227 | - | - |
|  | *trnG-UCC* | LSC | 23 | 744 | 48 | - | - |
|  | *atpF* | LSC | 145 | 797 | 416 | - | - |
|  | *rpoC1* | LSC | 432 | 800 | 1611 | - | - |
|  | *pafI* | LSC | 124 | 737 | 230 | 795 | 153 |
|  | *trnL-UAA* | LSC | 35 | 552 | 50 | - | - |
|  | *trnV-UAC* | LSC | 39 | 596 | 35 | - | - |
|  | *rps12** | LSC | - | 538 | 232 | - | 26 |
|  | *clpP1* | LSC | 71 | 872 | 292 | 593 | 228 |
|  | *petB* | LSC | 6 | 754 | 642 | - | - |
|  | *petD* | LSC | 8 | 739 | 475 | - | - |
|  | *rpl16* | LSC | 9 | 1039 | 399 | - | - |
|  | *rpl2* | IR | 391 | 693 | 434 | - | - |
|  | *ndhB* | IR | 777 | 681 | 756 | - | - |
|  | *trnI-GAU* | IR | 37 | 961 | 35 | - | - |
|  | *trnA-UGC* | IR | 38 | 803 | 35 | - | - |
|  | *ndhA* | SSC | 553 | 1153 | 539 | - | - |

Notes: LSC、SSC、IR refer to the large single-copy region、the small single-copy region and the inverted repeat region, respectively. *The *rps12* gene is a trans-spliced gene with the 5′ end located in the LSC region and the duplicated 3′ ends in the IR regions.
