# Supplementary material for: Comparative chloroplast genome analysis of five widespread species (Zanthoxylum L.) and development of molecular markers for their discrimination
Source: Front Genet. 2024 Dec 24;15:1495891. doi: 10.3389/fgene.2024.1495891 (PMC11703814; doi:10.3389/fgene.2024.1495891)
Supplement: Supplementary file 6 [file Table2.doc]

Table S2. Taxonomic and accession information for samples used in the study.

| Taxon | Order, Family | GenBank Number |
| --- | --- | --- |
| *Zanthoxylum bungeanum* 1 | Rutales, Rutaceae | MW206786 |
| *Zanthoxylum bungeanum* 2 | Rutales, Rutaceae | MW206787 |
| *Zanthoxylum bungeanum var. punctatum* | Rutales, Rutaceae | MW246146 |
| *Zanthoxylum piasezkii* 1 | Rutales, Rutaceae | MW206785 |
| *Zanthoxylum piasezkii* 2 | Rutales, Rutaceae | MW602888 |
| *Zanthoxylum piasezkii* 3 | Rutales, Rutaceae | MW602889 |
| *Zanthoxylum armatum* 1 | Rutales, Rutaceae | MW602887 |
| *Zanthoxylum armatum* 2 | Rutales, Rutaceae | MW602883 |
| *Zanthoxylum piperitum* | Rutales, Rutaceae | NC027939 |
| *Zanthoxylum simulans* | Rutales, Rutaceae | NC037482 |
| *Zanthoxylum motuoense* | Rutales, Rutaceae | MT990981 |
| *Zanthoxylum acanthopodium* | Rutales, Rutaceae | NC051878 |
| *Zanthoxylum nitidum* | Rutales, Rutaceae | MW602879 |
| *Zanthoxylum nitidum var. tomentosum* | Rutales, Rutaceae | MN241098 |
| *Zanthoxylum tragodes* | Rutales, Rutaceae | NC046747 |
| *Zanthoxylum dissitum* | Rutales, Rutaceae | NC068581 |
| *Zanthoxylum echinocarpum* | Rutales, Rutaceae | NC068579 |
| *Zanthoxylum scandens* | Rutales, Rutaceae | NC068579 |
| *Zanthoxylum multijugum* | Rutales, Rutaceae | MT990982 |
| *Zanthoxylum calcicola* | Rutales, Rutaceae | MT990983 |
| *Zanthoxylum oxyphyllum* | Rutales, Rutaceae | MT990980 |
| *Zanthoxylum stenophyllum* | Rutales, Rutaceae | NC058754 |
| *Zanthoxylum micranthum* | Rutales, Rutaceae | NC066647 |
| *Zanthoxvlum schinifolium* | Rutales, Rutaceae | NC030702 |
| *Zanthoxvlum avicennae* | Rutales, Rutaceae | NC062045 |
| *Zanthoxylum ailanthoides* | Rutales, Rutaceae | MW478808 |
| *Zanthoxylum dimorphophyllum* | Rutales, Rutaceae | MW478807 |
| *Zanthoxylum madagascariense* | Rutales, Rutaceae | NC046744 |
| *Zanthoxylum paniculatum* | Rutales, Rutaceae | NC046745 |
| *Phellodendron chinense* | Rutales, Rutaceae | MT916287 |
| *Tetradium ruticarpum* | Rutales, Rutaceae | MT800757 |
